# First Onset in Adulthood of Mental Disorders: Exposure to War *vs*. Non-war Childhood Adversities: A National Study

**DOI:** 10.2174/0117450179216651231106072824

**Published:** 2023-11-07

**Authors:** Elie Karam, Josleen Al Barathie, Dahlia Saab, Aimee Nasser Karam, John Fayyad

**Affiliations:** 1 Institute for Development, Research, Advocacy and Applied Care (IDRAAC), Beirut, Lebanon; 2 Department of Psychiatry and Clinical Psychology, University of Balamand Faculty of Medicine, Beirut, Lebanon; 3 Department of Psychiatry and Clinical Psychology, St George Hospital University Medical Center, Beirut, Lebanon

**Keywords:** Mood disorders, Anxiety disorders, Adult-onset, War exposure, Childhood adversities, Trauma, Family malfunctioning

## Abstract

**Background:**

There is evidence that some childhood trauma increases the risk of the first onset of mental disorders and for the first time into adulthood. There are no studies that assessed whether exposure to war has this delayed long-term effect.

**Objectives:**

To fill this gap by investigating the comparative roles of war and non-war trauma on the first onset of adulthood mood and anxiety disorders.

**Methods:**

A nationally representative sample of 2,857 Lebanese was assessed using the World Health Organization Composite International Diagnostic Interview 3.0. with the onset of exposure to trauma and of first onset of mood and anxiety disorders.

**Results:**

Non-war childhood traumata especially those belonging to family malfunctioning continue to exert their effect for the first time well beyond their occurrence as they were the most universal predictors for adult onset of both mood and anxiety disorders. War trauma during childhood predicted mood anxiety and mood (anxiety only in males) only below age 18 y. war childhood trauma predicts the first onset of mood and anxiety disorders before age 18 y in females, but only anxiety in males.

**Conclusion:**

Childhood traumata are not equal in predicting the first onset of mood and anxiety disorders into adulthood. Family malfunctioning looks to carry the longest such risk and war more of shorter immediate effects. This might change though with re-exposure to war in adulthood which might unravel dormant vulnerability.

## 1 INTRODUCTION

Historically, single types of childhood adversities were investigated when assessing their effect on developing lifetime mental disorders in adulthood. Some looked at the individual effects of childhood physical abuse, sexual abuse or neglect [1, 2]. Others investigated the differential effects of more than one adversity such as childhood maltreatment (abuse, neglect), parental substance use [3], or bullying [4].

More recently, research investigating this has expanded to include a larger variety of adversities using the World Mental Health (WMH) surveys including interpersonal loss (parental death, divorce, or separation), parental maladjustment (mental illness, substance misuse, criminality, and violence), as well as maltreatment (physical abuse, sexual abuse, and neglect), in addition to life-threatening respondent physical illness and family economic adversity [5]. It was found that the probable attributable risk of childhood adversities on first onset of adult disorders is 29.8% for all disorders across 21 countries, notably those related to maladaptive family functioning (including neglect, abuse, and family psychopathology) [5].

Studies exploring this wide range of childhood adversities and major collective large-scale trauma such as war on the first onset of mental disorders later in adulthood are lacking. More specifically, and in the case of the long-term impact of war exposure in childhood on the development of mental disorders, none to our knowledge, compared them to past non-war childhood other traumatic events [6, 7], let alone on the first onset of mental disorders in adulthood. Additionally, a lot of studies focused on very few or even one war event to predict mental disorders at a later stage in life. This is of major importance as evidenced by a report from our group including some of the co-authors showing that children and adolescents who were exposed to war were also more likely to be exposed to non-war childhood adversities [8]. One study investigated one traumatic war event focusing on the lasting impact of parental loss, specifically the death of a father during childhood or adolescence due to war-related violence, on the mental health and functioning of young adults. The findings indicate that individuals who experience this type of loss face a heightened risk of mental health issues and functional impairments during their young adulthood [9]. The few studies in the literature that we could find and which looked at both sets (war and non-war) were very short-term (one 4 years post-war in Northern Uganda [10] and one after one year in Kabul, Afghanistan [11]) and found both war and childhood adversities to be associated with negative consequences on mental health [10, 11]. Two additional studies from our group, where violence in the family emerged as a stronger predictor than war exposure in predicting the persistence of many mental health disorders, were also very short-term (one year) prospective studies. However, none of these studies addressed the issue of first onset in adulthood after war exposure [12, 13].

We had shown in a large national study The L.E.B.A.N.O.N study (Lebanese Evaluation of the Burden of Ailments and Needs of the Nation) [14] that exposure to war, in general, was associated with the first-ever onset of mood, anxiety, and externalizing disorders [15]. In this paper we try to fill a gap by investigating, in this same national study, the outcome of concomitant exposure, in childhood and adolescence to war and a wide range of non-war adverse traumatic events on first onset in adulthood (>18yrs) of mood and anxiety disorders.

## 2 MATERIALS AND METHODS

The data used in this paper is from the L.E.B.A.N.O.N. study conducted by the Institute for Development Research Advocacy and Applied Care (IDRAAC) in association with the Department of Psychiatry and Clinical Psychology at the University of Balamand, Faculty of Medicine and St. George Hospital University Medical Center (SGHUMC), and is part of the World Health Organization (WHO) WMH Survey Initiative conducted in association with the Harvard Medical School in the US. This study was approved by the Institutional Review Board (IRB) committee of the SGHUMC Faculty of Medicine, University of Balamand, Lebanon, which is registered with the U.S. Office of Human Research Protections (OHRP) in the Department of Health and Human Services.

### 2.1 Sample

A nationally representative sample of non-institutionalized Lebanese adults was selected using a stratified multi-stage cluster design with a probability proportionate to size approach. Details of the sample can be found elsewhere [14]. A total of 2,857 adults were interviewed with a response rate of 70%.

Interviews were conducted face-to-face by lay interviewers whom we trained using the Lebanese Arabic version of the WHO Composite International Diagnostic Interview (CIDI) Version 3.0 [2]. Details of the interviewers’ training and fieldwork are provided elsewhere [14]. All respondents (n=2,857) completed Part I of the CIDI which is an assessment of core mental disorders. Respondents who met the lifetime criteria for any core disorder plus a probability sub-sample of other respondents (n=1,031) were administered Part II of the CIDI which comprised other disorders and their correlates. Additional details about the sampling procedure have been reported elsewhere [14, 15].

For this study, only the Part II sample was analyzed as only this part included an assessment of exposure to childhood adversities (CAs) and traumatic events (TEs). The Part II sample was weighted for differential probability of selection to achieve national representativeness.

### 2.2 Measures

The CIDI version 3.0 was translated from English to Arabic and the following measures were created [14].

#### 2.2.1 Mental Disorders

Anxiety and mood disorders were assessed according to the Diagnostic Statistical Manual IV (DSMIV) criteria. Mood disorders included: Major Depressive Disorder (MDD), dysthymia, and Bipolar Disorder (BP). Anxiety disorders included: Agoraphobia without panic, Social phobia, Specific phobia, Panic disorder, Post-Traumatic Stress Disorder (PTSD), Generalized Anxiety Disorder (GAD), and Separation anxiety disorder. Impulse control disorder (first onset >18 years=10) and Substance use disorders were not included in the analyses due to small sample sizes (with n=8 and 27 respectively).

#### 2.2.2 Childhood Traumatic Events (CTEs)

CTEs were assessed in the PTSD section of the CIDI. Respondents were asked about the age of onset of each event. A “childhood traumatic event” (CTE) was defined as one that has occurred before the age of 18 years. A follow-up question asked whether this specific event was related to war or not. Accordingly, events were further divided into war and non-war CTEs. Additional non-war CTEs that were not included in the PTSD/ trauma section of the CIDI, were also assessed in a separate childhood experiences section referring to the period before the age of 16 years and were added to the list of non-war CTEs.

##### 2.2.2.1 *War CTEs* were Grouped into four Categories

*Active war exposure*: involvement in combat, volunteer/rescue worker in a war zone, and intentionally injuring/ torturing/ or killing someone.

##### 2.2.2.2 General war Exposure

Civilians in war zones, civilians in terror zones, and refugees.

##### 2.2.2.3 Direct personal trauma

kidnapped, threatened at gunpoint/ robbery, stalked, physical abuse from another person, and life-threatening accident.

##### 2.2.2.4 Trauma to others related directly to war

Traumatic event to a loved one, witnessing death/ dead body/ severe injury, witnessing atrocities, and unexpected death to a loved one (excluding parent death as all parental deaths were treated as non-war CTEs since we could not, unfortunately, separate them).

##### 2.2.2.5 *Non-war CTEs* were Grouped into Eight Categories

*Direct personal trauma*: physical abuse from the spouse (we had 7 participants who were married before the age of 18 years, physical abuse from another person (other than parents or caregivers), sexual abuse (happening once or twice), and unspecified private events.

##### 2.2.2.6 Other Personal Trauma

Threatened at gunpoint/robbery, stalked, car accident, life-threatening accident.

##### 2.2.2.7 Trauma to others

Traumatic event to a loved one, witnessing death/ dead body/ severe injury, witnessing non-war atrocities, manmade disaster other than war, natural disaster, and the unexpected death of a loved one (excluding parent death which is categorized separately under parental loss).

##### 2.2.2.8 Caused harm to Someone

Accidentally caused serious injury/ death, and intentionally injuring/ torturing/ killing someone outside a war context.

##### 2.2.2.9 Neglect and Abuse

Neglect, physical abuse from parents or caregivers, and sexual abuse (happening 3 or more times).

##### 2.2.2.10 Parental Loss

Parent death and change in family structure.

##### 2.2.2.11 Parent Psychopathology

Mental or substance use disorder, family violence, and criminal behavior.

##### 2.2.2.12 Other Adversities

Family economic adversity and having a life-threatening illness/chronic medical condition during childhood.

### 2.3 Outcomes

The two outcomes of interest are the onset of mood and anxiety disorders. The outcomes are classified into three categories: No disorder, onset of disorder below age 18 y, and onset of disorder above 18 y.

### 2.4 Statistical Analyses

First, bivariate logistic regression models were built to assess the association of the following outcomes: adult, above age 18 y onset of “any mood disorder” and “any anxiety disorder” and onset of same disorders before age 18 y with: each of the four categories of the war CTEs listed above, and each of the eight categories of non-war CTEs. Then, multivariable logistic regression models (adjusting for gender) were created keeping variables that maintained a statistical significance at the alpha level of <0.05. Crude and adjusted Odds Ratios (OR) with their 95% Confidence Intervals (CIs) were generated. Furthermore, the same analyses were repeated while stratifying our sample by males and females respectively. All logistic regression models were weighted to account for differential sample selection and subsampling for specific questionnaire sections. The data were analyzed using the program Stata version 13.

## 3 RESULTS

### 3.1 Description of Exposure to War and Non-war CTEs in the Total Sample and by Gender

The most common exposure to children and adolescents as reported was being a civilian in a war zone (N=292, 28.97%) followed by being a refugee (173, N=17.2%), death of a parent (142, N=12.9%), and having a parent with a mental health disorder (148, N=11.14%). For more information about the descriptive data, please refer to Figs. (**1** and **2**).

### 3.2 First Onset in Adulthood of Mood Disorders: Bivariate Level

Table **1** provides an overview of the two main categories of predictors: War Childhood Traumatic Experiences (War CTEs) and Non-War Childhood Traumatic Experiences (Non-War CTEs). These predictors are examined with the onset of adult mood disorders *versus* the absence of mood disorders at the bivariate level.

None of the War CTEs were significant predictors of the first onset of adult mood disorders in the total sample. After stratifying by gender and at the bivariate level, general war exposure was a significant predictor for males (OR: 2.08; 95%CI [1.04 – 4.14]), while none of the war CTEs were significant predictors for first adult mood onset for females.

With regards to Non-War CTEs and at the bivariate level, in the total sample, neglect and abuse (OR: 3.97; 95%CI [2.03 – 7.78]) and parent psychopathology (OR: 3; 95%CI [ 1.81- 4.95]) were significantly associated with first adult mood onset. When stratifying by gender and at the bivariate level, neglect, and abuse (for males: OR: 5.18; 95%CI [1.87 – 14.33]/ for females (OR: 3.29; 95%CI [1.34 – 8.09]) and parent psychopathology (for males: OR: 3; 95%CI [1.27 – 7.11]/ for females: (OR: 2.77; 95%CI [1.48 – 5.17]) were two consistent predictors of adult mood first onset for both females and males, separately. A rather surprising finding and only in the male sample, was the finding that parental loss showed as a borderline significantly protective factor (OR: 0.27; 95%CI [0.07 – 1.07]) for adult mood first onset at the bivariate levels.

### 3.3 First Onset in Adulthood of Mood Disorders: Multivariable Level

For War CTEs (Table **1**), at the multivariable level, general war exposure remained borderline significant in the male sample (OR: 1.86; 95%CI [0.91 – 3.9]) (Table **1**).

For Non-War CTEs (Table **1**), in the total sample, and at the multivariable level, neglect and abuse (OR: 2.9; 95%CI [1.42- 5.94]) and parent psychopathology (OR: 2.33; 95%CI [1.37 – 3.98]) remained significant. When stratifying by gender, neglect and abuse remained significant in males (OR: 4.54; 95%CI [ 1.42- 14.57]), but borderline in females (OR: 2.45; 95%CI [0.96 -6.28]). However, parent psychopathology remained a significant predictor only in the female sample (OR: 2.43; 95%CI [1.27 – 4.64]) and was borderline in males (OR: 2.45; 95%CI [0.94 – 6.35]). Similarly, to the bivariate level and in males only, parental loss remained a significant protective factor for adult mood onset (OR: 0.19; 95%CI [0.04 – 0.81]).

### 3.4 First Onset in Adulthood of Anxiety Disorders: Bivariate Level

Table **2** summarizes the two broad categories of predictors (War CTEs and Non-War CTEs) of adult anxiety onset compared to no anxiety at the bivariate level. Only active war exposure among the war CTEs was a borderline significant (OR: 3.61; 95%CI [ 0.9- 14.55]) predictor of the first onset of adult anxiety disorders in the total sample. After stratifying by gender and at the bivariate level, war CTEs were neither significant predictors in males nor in females of first adult anxiety onset.

With regards to Non-War CTEs and at the bivariate level, in the total sample, neglect, and abuse was significant predictor (OR: 2.86; 95%CI [ 1.02- 7.99]) of first adult anxiety onset. However, parent psychopathology was borderline significant (OR: 2.04; 95%CI [ 0.91- 4.59]). When stratifying by gender and at the bivariate level, neglect and abuse (OR: 5.14; 95%CI [ 1.06- 25.07]) was a consistent predictor of adult anxiety first onset for males only while parental loss (OR: 2.39; 95%CI [ 1.02- 5.61]) appeared as a significant predictor in females only.

### 3.5 First Onset in Adulthood of Anxiety Disorders: Multivariable Level

For War CTEs (Table **2**), at the multivariable level, active war exposure was the only predictor that remained significant (OR: 6.16; 95%CI [ 1.34- 28.38]) in the total sample.

For non-war CTEs (Table **2**), when stratifying by gender, neglect and abuse remained the only significant abuse (OR: 5.14; 95%CI [ 1.06- 25.07]) in males and parental loss was the only significant predictor (OR: 2.39; 95%CI [ 1.02- 5.61]) in females.

### 3.6 First onset of mood disorders before age 18y: Bivariate level. Please see supplementary table **1** and supplementary text **1**.

### 3.7 First onset of mood disorders before 18y: Multivariable level. Please see supplementary table **1**.

For War CTEs (Table **S1**), at the multivariable level, only general war exposure was shown to be borderline significant for the total sample (OR: 2.12; 95%CI [ 0.96- 4.66]). For males, none of the war CTEs was a significant predictor of the onset of mood disorders before 18y at the multivariate level. When looking at females, general war exposure remained significant (OR: 3.18; 95%CI [ 1- 10.11]) while trauma to others turned out to be borderline significant (OR: 3.26; 95%CI [ 0.94- 11.23]).

For Non-war CTEs (Table **S1**), at the multivariate level, in the total sample, direct trauma to others (OR: 3.83; 95%CI [ 1.35- 10.86]) and neglect and abuse (OR: 3.31; 95%CI [ 1.24- 8.82]) remained significant while caused harm to someone was borderline significant (OR: 11.29; 95%CI [ 0.83- 152.8]). When looking at non-war CTEs in males at the multivariate level, only other adversities within the family remained significant (OR: 5.33; 95%CI [ 1.07- 26.54]). As for females, direct personal trauma (OR: 10.6; 95%CI [2.34 – 48.02]) and other personal trauma (OR: 7.61; 95%CI [1.27 – 48.46]) remained significant predictors of the onset of mood disorders before 18y.

### 3.8 First onset of anxiety disorders before age 18y:

Bivariate level. Please see supplementary table 2 and supplementary text 2.

### 3.9 First onset of anxiety disorders before 18y: Multivariable level. Please see supplementary table **2**.

For war CTEs (Table **S2**), at the multivariable level, only general war exposure remained significant for the total sample (OR: 1.62; 95%CI [ 1.09- 2.4]). For both males and females, general war exposure (for males: OR: 1.89; 95%CI [ 0.89- 4]/ for females: (OR: 1.49; 95%CI [ 0.93- 2.38]) was a borderline significant predictor of the onset of anxiety disorders before 18y at the multivariate level.

For non-war CTEs (Table **S2**), at the multivariate level, in total sample, trauma to others (OR: 2.27; 95%CI [ 1.41- 3.67]), parent psychopathology (OR: 2.23; 95%CI [ 1.34- 3.74]) and neglect and abuse (OR: 2.02; 95%CI [ 0.99- 4.09]) remained significant predictors of first onset of anxiety below 18y, yet the latter was borderline significant. When looking at non-war CTEs in males at the multivariate level, parent psychopathology (OR: 2.7; 95%CI [ 1.04- 6.99]) and trauma to other (OR: 2.35; 95%CI [ 0.98- 5.65]) were significant predictors, yet the latter was borderline. As for females, trauma to others (OR: 2.18; 95%CI [ 1.23- 3.86]), parent psychopathology (OR: 1.91; 95%CI [ 1.03- 3.36]) and neglect and abuse (OR: 2.19; 95%CI [ 0.93- 5.18]) were significant with the latter being a borderline significant predictor of the onset of anxiety disorders before 18y.

## 4 DISCUSSION

Negative life events in childhood have been repeatedly shown to be associated with mental disorders in adulthood [10, 16] and the challenge remains to cover all negative events. The mechanisms underlying the associations are not well understood and are thought by some to be essential primers interacting with the genetic makeup of individuals early on in life [17, 18]. All too commonly, one life event or a group of events has been singled out to study this robust association. The most commonly studied events are referred to as “childhood adversities”. Another category of negative events belongs to the general category of “traumatic events”. War brings an additional series of traumatic events that can further impact the growing brains of children and adolescents. We had previously found that war events were significantly associated not only with first onset of mental disorders but also with their severity [14, 15, 19]. In a previous analysis of the data from the same sample, we had explored the effect of cumulative war exposure on first onset of mental disorders and found that war was a significant predictor of mental health disorders, particularly of mood and anxiety which are the focus of the current analysis (respective ORs [95% CIs]: 3.32 [2.0-5.6], 5.92 [2.5-14.1]) [15].

Studying war and non-war-related traumatic events is obviously imperative since events from categories seem to co-occur more in specific settings (eg: families, and neighborhoods) [8]. To our knowledge, this is the first published national study assessing simultaneously the relation of childhood exposure to war as well as that of non-war adversities to the *first onset* of mental disorders mainly mood and anxiety. Also, previous research did not assess specific events with types of disorders [20]. In addition, the analysis in previous research did not examine specific patterns in males *vs*. females. In this study, we focused on the stratification by gender and the differences in the effect of war and non-war CTEs on the first onset of the disorders.

The results of this study should be interpreted in the light of some limitations. First, as data were collected through face-to-face interviews, social desirability bias may have resulted in the underreporting of adversities and traumas. Second, recall bias could also have led to the under-reporting of symptoms of mental disorders. The recall of war and non-war traumata may have been biased by reporting on events that occurred many years or decades prior to the interview. Also, there are no studies that compare the differential recall between war and non-war traumatic events. Thirdly, we could not study the temporality of exposures and onset of mental disorders below age 18 since we do not have data on the exact “onset” of childhood events before age 18 years. Fourthly, we have not measured psychotic disorders, nor impulse and substance use disorders. Fifthly, the sample size might have limited some of our findings. Sixthly, the sample did not include institutionalized respondents. Seventhly, not all specific war events are represented in this population of childhood exposure such as kidnapping which we had found to be linked to severe and protracted consequences and to be linked to the longest PTSD of all war traumata and if it is present, it might have had different effects [21]. Eight, childhood adversities were measured only till age 16y so there might have been other adversities between 16y and 18y and if we had measured them, they could have increased the importance of non-war relative to war. Nine, the death of parents due to war could not be put under war adversities and thus it is possible that this single exception might have shown a delayed effect on the first onset of disorders. Lastly, we did not measure for severity, frequency, and recurrence of the war and non-war variables, so we could not account for this as the non-war (happening inside the family) could be a chronic exposure while the war exposure could be a one-time event or of a less frequent nature.

Notwithstanding the above limitations, when all the childhood traumatic events were analyzed together in multivariable models, it is clear that the most universal predictor for adult onset of both mood and anxiety disorders were the non-war traumata, and within those, more specifically “neglect and abuse”. This is in line with Kessler *et al*, who have found that maladaptive family functioning is clearly the most important predictor, although not the only one; still its effect on first onset is much stronger early on in life than first onset later on [5].

War trauma, by contrast, predicted (significant at bivariate and borderline at the multivariable level) the first onset after age 18 years of mood disorders only in males. Although its effect lost practically its importance compared to non-war trauma, when we put them together in a multivariable model, it was still present.

Moreover, when we looked at the first onset of disorders before age 18 y, war exposure was related to mood and anxiety disorders in females, but only to anxiety in males. This is important because it implies that the effect of traumatic events might not be equal for all these events over *time*. It is probable that some events (such as the case of war in females) exerted their effects on first onset of mood disorders early on after exposure and thus did not have a delayed effect later in life. Whereas the more powerful non-war CTEs exerted their effect on both mood and anxiety for the first time ever before 18y, their effect continued to unravel at a later stage after 18y as shown elsewhere [5]. It is important to acknowledge here the possibility that re-exposure to war in adulthood (and other disasters) might unravel an increased risk that lay “dormant” such as the case of PTSD.

The reason why some traumata continue to uncover their effects long after the exposure while others do not is an important issue that this research was not designed to explain. Of course, there are many reasons affecting first onset of mood or anxiety disorders including genetics and other temperamental characteristics [22] but everything else (non-war CTEs) being equal, war exposure seems to exert its effect mostly on the first onset before age 18y in females and after 18y in males. Also, it is possible that one of the childhood adversities, parental psychopathology, has by definition long term effects that are not the result of “direct” trauma only but also in determining important lifelong genetic and behavioral liabilities.

These findings are interesting clinically at the individual and collective levels; thus, when waving the history of a society (in this case, Lebanon) or an individual the effect of war, especially its relative weight to other traumata, the potential effect of each takes on a different dimension on a personal or collective level. It would be interesting to see this work replicated in the setting of other major disasters including wars and confirm whether the hard-core non-war childhood adversities remain the most important predictors of ^the^ first onset of internalizing disorders in adulthood.

## CONCLUSION

In summary, our study reveals that war-related childhood traumatic experiences (CTEs) were not significant predictors of mood disorder onset among the total sample and males. However, among females, general war exposure was a predictor for mood onset below 18. Notably, non-war CTEs, specifically neglect and abuse, remained significant predictors in the total sample in below 18 onsets and adult onset, emphasizing the enduring impact of these experiences on mental health outcomes. The variability in other predictors by gender between below 18 and adult-onset was discussed above. As for anxiety onset, our study reveals that different war-related CTEs predict the onset of anxiety disorders at different age intervals. Active war exposure predicts adult-onset, while general war exposure is linked to onset below 18, with no gender-specific effects. Among non-war CTEs, parental loss predicts adult anxiety in females, neglect and abuse predicts adult anxiety in males, while various factors contribute to onset below 18, including parent psychopathology for the total sample and both genders and trauma to others for the total sample and female.

Consequently, childhood traumata are not equal in predicting the first onset of mood and anxiety disorders neither in adulthood or below 18. Family malfunctioning of the non-war CTEs looks to carry the longest such risk and war more of shorter immediate effects. This might change though with re-exposure to war in adulthood which might unravel dormant vulnerability.

## Figures and Tables

**Fig. (1):**
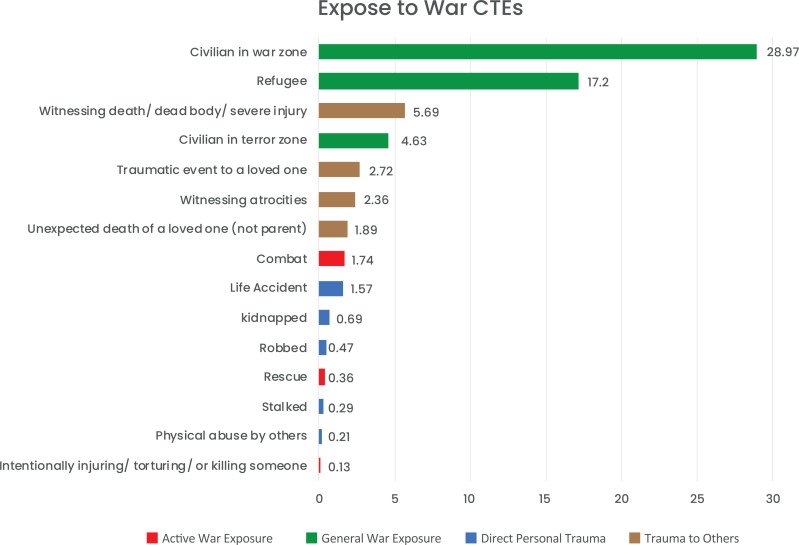
Exposure to war CTE by type.

**Fig. (2):**
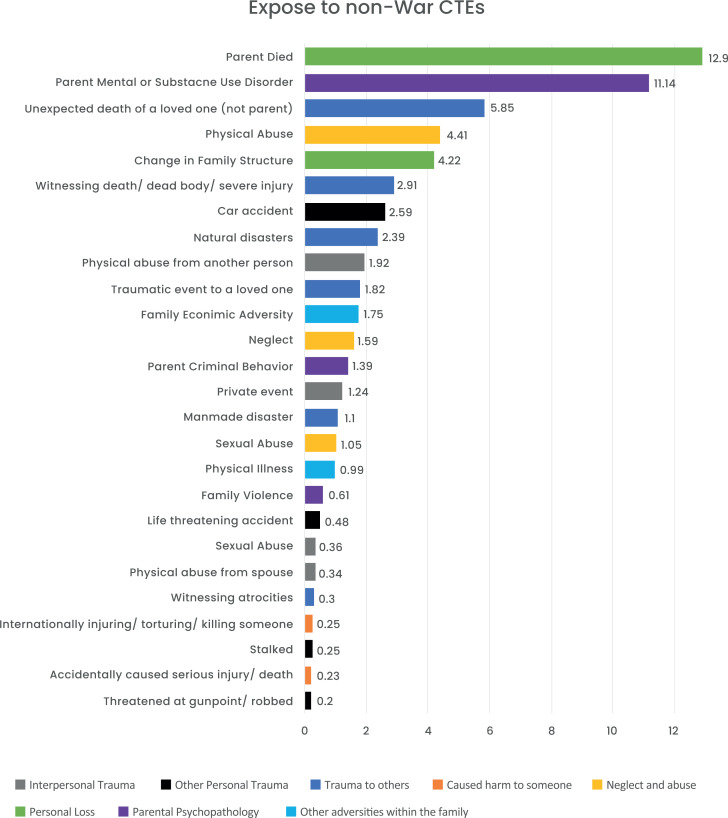
Exposure to non-war CTE by type.

**Table 1:** War and non-war trauma as predictors of adult first onset of mood disorders^ *vs*. no mood disorder at the bivariate and multivariable level.

-	-	Total Sample (n=960) Bivariate				Adult Males (n=402) Bivariate				Adult Females (n=558) Bivariate			
		OR	95%		P-value	OR	95%		P-value	OR	95%		P-value
			Confidence Limits				Confidence Limits				Confidence Limits		
War CTEs	Active War Exposure	1.19	0.29	4.89	0.807	1.59	0.35	7.31	0.547	2.32	0.03	188.22	0.707
	General War Exposure	1.3	0.85	1.99	0.233	2.08	1.04	4.14	0.038*	0.92	0.53	1.61	0.771
	Direct Personal Trauma	0.57	0.11	3	0.503	0.99	0.15	6.76	0.994	0.26	0.01	8.67	0.453
	Trauma to Others	1.18	0.6	2.33	0.625	1.88	0.78	4.53	0.158	0.83	0.27	2.53	0.739
Non War CTEs	Direct Personal Trauma	1.93	0.76	4.91	0.166	1.09	0.16	7.47	0.292	2.41	0.8	7.28	0.119
	Other Personal Trauma	2.1	0.8	5.53	0.134	2.71	0.77	9.55	0.121	1.98	0.42	9.35	0.389
	Trauma to Others	1.59	0.92	2.75	0.097	1.8	0.73	4.41	0.199	1.42	0.71	2.85	0.326
	Caused Harm to Someone	4.5	0.49	41.07	0.183	4.76	0.4	56.71	0.218	-	-	-	-
	Neglect & Abuse	3.97	2.03	7.78	<0.001*	5.18	1.87	14.33	0.002*	3.29	1.34	8.09	0.009*
	Parental Loss	0.74	0.4	1.37	0.334	0.27	0.07	1.07	0.063~	1.31	0.64	2.71	0.462
	Parent Psychopathology	3	1.81	4.95	<0.001*	3	1.27	7.11	0.012*	2.77	1.48	5.17	0.001*
	Other Adversities within the Family	1.19	0.34	4.13	0.786	0.32	0.01	12.37	0.544	1.76	0.44	7.05	0.427
-	Sex (M vs F)	0.53	0.35	0.82	0.005*	-	-	-	-	-	-	-	-

Note: *: p<0.05.

~: p<0.1.

^ Major Depressive Disorder (MDD), dysthymia, and Bipolar Disorder (BP).

**Table:** 

-	-	Total Sample (n=960) Multivariate				Adult Males (n=402) Multivariate				Adult Females (n=558) Multivariate			
		OR	95%		P-value	OR	95%		P-value	OR	95%		P-value
			Confidence Limits				Confidence Limits				Confidence Limits		
War CTEs	Active War Exposure	-	-	-	-	-	-	-	-	-	-	-	-
	General War Exposure	-	-	-	-	1.86	0.91	3.9	0.091~	-	-	-	-
	Direct Personal Trauma	-	-	-	-	-	-	-	-	-	-	-	-
	Trauma to Others	-	-	-	-	-	-	-	-	-	-	-	-
Non War CTEs	Direct Personal Trauma	-	-	-	-	-	-	-	-	-	-	-	-
	Other Personal Trauma	-	-	-	-	-	-	-	-	-	-	-	-
	Trauma to Others	-	-	-	-	-	-	-	-	-	-	-	-
	Caused Harm to Someone	-	-	-	-	-	-	-	-	-	-	-	-
	Neglect & Abuse	2.9	1.42	5.94	0.004*	4.54	1.42	14.57	0.011*	2.45	0.96	6.28	0.062~
	Parental Loss	-	-	-	-	0.19	0.04	0.81	0.025*	-	-	-	-
	Parent Psychopathology	2.33	1.37	3.98	0.002*	2.45	0.94	6.35	0.065~	2.43	1.27	4.64	0.007*
	Other Adversities within the Family	-	-	-	-	-	-	-	-	-	-	-	-
-	Sex (M vs F)	0.55	0.36	0.86	0.008*	-	-	-	-	-	-	-	-

Note: *: p<0.05.

~: p<0.1.

^ Major Depressive Disorder (MDD), dysthymia, and Bipolar Disorder (BP).

**Table 2:** War and non-war childhood trauma as predictors of adult first onset of anxiety disorders^^ *vs*. no anxiety disorder at the bivariate and multivariable level.

-	-	Total Sample (n=846) Bivariate				Adult Males (n=374) Bivariate				Adult Females (n=472) Bivariate			
		OR	95%		P-value	OR	95%		P-value	OR	95%		P-value
			Confidence Limits				Confidence Limits				Confidence Limits		
War CTEs	Active War Exposure	3.61	0.9	14.55	0.071~	4.47	0.69	28.82	0.115	-	-	-	-
	General War Exposure	1.03	0.54	1.99	0.921	1.64	0.49	5.5	0.426	0.79	0.36	1.75	0.565
	Direct Personal Trauma	0.53	0.04	7.73	0.641	1.98	0.13	31.24	0.627	-	-	-	-
	Trauma to Others	1.32	0.51	3.43	0.564	2.05	0.48	8.82	0.337	1.35	0.67	4.99	0.65
Non War CTEs	Direct Personal Trauma	1.09	0.21	5.64	0.916	2.33	0.22	24.58	0.481	0.67	0.07	6.75	0.733
	Other Personal Trauma	0.7	0.08	6	0.748	1.42	0.09	22.08	0.802	0.47	0.01	15.65	0.673
	Trauma to Others	1.18	0.47	2.98	0.717	1.52	0.29	8.01	0.623	1.02	0.34	3.17	0.968
	Caused Harm to Someone	-	-	-	-	-	-	-	-	-	-	-	-
	Neglect & Abuse	2.86	1.02	7.99	0.046*	5.14	1.06	25.07	0.043*	2.15	0.54	8.61	0.277
	Parental Loss	1.5	0.71	3.18	0.289	0.49	0.07	3.56	0.479	2.39	1.02	5.61	0.044*
	Parent Psychopathology	2.04	0.91	4.59	0.085~	2.99	0.69	12.88	0.141	1.58	0.59	4.22	0.364
	Other Adversities within the Family	1.82	0.42	7.98	0.426	1.54	0.07	34.29	0.786	1.85	0.33	10.19	0.482
-	Sex (M *vs* F)	0.3	0.15	0.61	0.001*	-	-	-	-	-	-	-	-

Note: *: p<0.05.

~:  p<0.1.

^^  Agoraphobia without panic, social phobia, specific phobia, panic disorder, Post-Traumatic Stress Disorder (PTSD), Generalized Anxiety Disorder (GAD), and separation anxiety disorder.

**Table:** 

-	-	Total Sample (n=846) Multivariate				Adult Males (n=374) Multivariate				Adult Females (n=472) Multivariate			
		OR	95%		P-value	OR	95%		P-value	OR	95%		P-value
			Confidence Limits				Confidence Limits				Confidence Limits		
War CTEs	Active War Exposure	6.16	1.34	28.38	0.02*	-	-	-	-	-	-	-	-
	General War Exposure	-	-	-	-	-	-	-	-	-	-	-	-
	Direct Personal Trauma	-	-	-	-	-	-	-	-	-	-	-	-
	Trauma to Others	-	-	-	-	-	-	-	-	-	-	-	-
Non War CTEs	Direct Personal Trauma	-	-	-	-	-	-	-	-	-	-	-	-
	Other Personal Trauma	-	-	-	-	-	-	-	-	-	-	-	-
	Trauma to Others	-	-	-	-	-	-	-	-	-	-	-	-
	Caused Harm to Someone	-	-	-	-	-	-	-	-	-	-	-	-
	Neglect & Abuse	2.41	0.8	7.27	0.117	5.14	1.05	25.07	0.043*	-	-	-	-
	Parental Loss	-	-	-	-		-	-	-	2.39	1.02	5.61	0.044*
	Parent Psychopathology	1.51	0.64	3.57	0.35		-	-	-	-	-	-	-
	Other Adversities within the Family	-	-	-	-		-	-	-	-	-	-	-
-	Sex (M *vs* F)	0.26	0.12	0.54	<0.001*		-	-	-	-	-	-	-

Note: *: p<0.05.

~: p<0.1.

^^ Agoraphobia without panic, social phobia, specific phobia, panic disorder, Post-Traumatic Stress Disorder (PTSD), Generalized Anxiety Disorder (GAD), and separation anxiety disorder.

## Data Availability

Availability of data will be studied with WMH group at Harvard as per prior agreement.

## LIST OF ABBREVIATIONS

WMH: World Mental Health
IDRAAC: Institute for Development Research Advocacy and Applied Care
SGHUMC: St. George Hospital University Medical Center
WHO: World Health Organization
CIDI: Composite International Diagnostic Interview
OHRP: Office of Human Research Protections
CAs: Childhood Adversities
TEs: Traumatic Events
DSMIV: Diagnostic Statistical Manual IV
MDD: Major Depressive Disorder
BP: Bipolar Disorder
PTSD: Post-traumatic Stress Disorder
GAD: Generalized Anxiety Disorder
CTEs: Childhood Traumatic Events
