# Supplementary material for: First Onset in Adulthood of Mental Disorders: Exposure to War vs. Non-war Childhood Adversities: A National Study
Source: Clin Pract Epidemiol Ment Health. 2023 Nov 7;19:e17450179216651. doi: 10.2174/0117450179216651231106072824 (PMC11037514; doi:10.2174/0117450179216651231106072824)
Supplement: Supplementary file 1 [file CPEMH-19-E17450179216651_SD1.pdf]

1745-0179/23

Send Orders for Reprints to reprints@benthamscience.net

Clinical Practice & Epidemiology in Mental Health

Content list available at: https://clinical-practice-and-epidemiology-in-mental-health.com

Supplementary Material

First Onset in Adulthood of Mental Disorders: Exposure to War *vs.* Non-war Childhood Adversities: A National Study

Elie Karam[1,2,3,*], Josleen Al Barathie[1], Dahlia Saab[1], Aimee Nasser Karam[1,2,3,#] and John Fayyad[1,2,3,ψ]

[1]*Institute for Development, Research, Advocacy and Applied Care (IDRAAC), Beirut, Lebanon*
[2]*Department of Psychiatry and Clinical Psychology, University of Balamand Faculty of Medicine, Beirut, Lebanon*
[3]*Department of Psychiatry and Clinical Psychology, St George Hospital University Medical Center, Beirut, Lebanon*

Supplementary Table 1. War and non-war trauma as predictors of onset below age 18 y of mood disorders^ *vs.* no mood disorders at the bivariate and multivariable level.

-	-	Total Sample (n=760) Bivariate				Males (n=344) Bivariate				Females (n=416) Bivariate			
-	-	OR	95% Confidence Limits		P-value	OR	95% Confidence Limits		P-value	OR	95% Confidence Limits		P-value
War CTEs	Active War Exposure	1.25	0.14	10.98	0.843	1.41	0.14	14.46	0.77	2.34	0.002	2345.1	0.809
	General War Exposure	3.54	1.78	7.05	<0.001*	2.39	0.87	6.53	0.09~	4.88	1.79	12.24	0.002*
	Direct Personal Trauma	2.5	0.63	9.96	0.194	-	-	-	-	6.29	1.38	28.62	0.017*
	Trauma to Others	3.28	1.51	7.14	0.003*	2.03	0.58	7.07	0.265	5.28	1.91	14.61	0.001*
Non War CTEs	Direct Personal Trauma	9.2	3.88	21.8	<0.001*	5.76	1.32	24.1	0.02*	12.17	4.06	36.48	<0.001*
	Other Personal Trauma	4.09	1.29	12.97	0.017*	2.66	0.43	16.29	0.291	6.92	1.46	32.75	0.015*
	Trauma to Others	1.79	0.78	4.1	0.17	1.35	0.32	5.63	0.68	2.05	0.73	5.76	0.174
	Caused Harm to Someone	14.35	1.72	119.56	0.014*	8.47	0.54	131.53	0.127	-	-	-	-
	Neglect & Abuse	6.94	2.95	16.36	<0.001*	7.4	2.04	26.91	0.002*	6.53	2.07	20.58	0.001*
	Parental Loss	1.47	0.67	3.25	0.338	1.3	0.4	4.18	0.66	1.81	0.61	5.34	0.284
	Parent Psychopathology	3.41	1.61	7.23	0.001*	4.51	1.46	13.93	0.009*	2.68	0.98	7.32	0.055~
	Other Adversities within the Family	3.6	1.04	12.43	0.043*	6.26	1.36	28.79	0.019*	1.72	0.18	16.48	0.639
-	Sex (M *vs* F)	0.71	0.37	1.39	0.323	-	-	-	-	-	-	-	-

Note: *: p<0.05.
~: p<0.1.
^ Major Depressive Disorder (MDD), dysthymia, and Bipolar Disorder (BP).

-	-	Total Sample (n=760) Multivariate				Males (n=344) Multivariate				Females (n=416) Multivariate			
-	-	OR	95% Confidence Limits		P-value	OR	95% Confidence Limits		P-value	OR	95% Confidence Limits		P-value
War CTEs	Active War Exposure	-	-	-	-	-	-	-	-	-	-	-	-
	General War Exposure	2.12	0.96	4.66	0.061~	1.91	0.61	5.96	0.262	3.18	1	10.11	0.049*
	Direct Personal Trauma	-	-	-	-	-	-	-	-	0.79	0.1	6.13	0.819
	Trauma to Others	1.52	0.59	3.94	0.388	-	-	-	-	3.26	0.94	11.23	0.061~

contd.....

-	-	Total Sample (n=760) Multivariate				Males (n=344) Multivariate				Females (n=416) Multivariate			
-	-	OR	95% Confidence Limits		P-value	OR	95% Confidence Limits		P-value	OR	95% Confidence Limits		P-value
Non War CTEs	Direct Personal Trauma	3.83	1.35	10.86	0.012*	1.79	0.31	10.37	0.516	10.6	2.34	48.02	0.002*
	Other Personal Trauma	1.69	0.41	6.94	0.468	-	-	-	-	7.61	1.27	48.46	0.026*
	Trauma to Others	-	-	-	-	-	-	-	-	-	-	-	-
	Caused Harm to Someone	11.29	0.83	152.8	0.068~	-	-	-	-	-	-	-	-
	Neglect & Abuse	3.31	1.24	8.82	0.017*	3.29	0.74	14.68	0.119	3.09	0.78	12.19	0.106
	Parental Loss	-	-	-	-	-	-	-	-	-	-	-	-
	Parent Psychopathology	1.26	0.5	3.16	0.625	2.73	0.78	9.54	0.115	0.5	0.12	2.11	0.35
	Other Adversities within the Family	2.09	0.51	8.48	0.304	5.33	1.07	26.54	0.041*	-	-	-	-
-	Sex (M *vs* F)	0.7	0.34	1.45	0.338	-	-	-	-	-	-	-	-

Note: *: p<0.05.
>~: p<0.1.
>^ Major Depressive Disorder (MDD), dysthymia, and Bipolar Disorder (BP).

Supplementary Table 2. War and non-war trauma as predictors of onset below age 18 y of anxiety disorders^^ *vs*. no anxiety disorders at the bivariate and multivariable level.

-	-	Total Sample (n=939) Bivariate				Males (n=404) Bivariate				Females (n=535) Bivariate			
-	-	OR	95% Confidence Limits		P-value	OR	95% Confidence Limits		P-value	OR	95% Confidence Limits		P-value
War CTEs	Active War Exposure	1.5	0.45	5.01	0.507	3.43	0.99	11.91	0.053~	-	-	-	-
	General War Exposure	2.02	1.4	2.92	<0.001*	2.24	1.11	4.52	0.024*	1.77	1.13	2.77	0.012*
	Direct Personal Trauma	1.79	0.7	4.56	0.226	4.52	1.33	15.38	0.016*	0.91	0.21	4.14	0.905
	Trauma to Others	1.33	0.76	2.35	0.321	1.33	0.5	3.54	0.563	1.77	0.84	3.7	0.131
Non War CTEs	Direct Personal Trauma	2.36	1.13	4.93	0.022*	1.79	0.38	8.5	0.464	2.39	0.99	5.77	0.052~
	Other Personal Trauma	1.55	0.63	3.84	0.342	2.83	0.82	9.81	0.101	1.27	0.32	4.97	0.731
	Trauma to Others	2.62	1.68	4.09	<0.001*	2.95	1.3	6.7	0.01*	2.38	1.38	4.12	0.002*
	Caused Harm to Someone	3	0.45	20.03	0.259	3.92	0.35	46.57	0.266	8.93	0.13	613.2	0.31
	Neglect & Abuse	3.07	1.64	5.75	<0.001*	3.06	0.99	9.47	0.053~	3.15	1.42	7.01	0.005*
	Parental Loss	1.05	0.64	1.71	0.856	2.1	0.98	4.47	0.055~	0.84	0.42	1.67	0.621
	Parent Psychopathology	3.03	1.93	4.76	<0.001*	3.81	1.66	8.71	0.002*	2.5	1.44	4.33	0.001*
	Other Adversities within the Family	0.86	0.26	2.86	0.808	2.34	0.5	11	0.283	0.39	0.06	2.77	0.349
-	Sex (M *vs* F)	0.29	0.19	0.43	<0.001*	-	-	-	-	-	-	-	-

Note: *: p<0.05.
~: p<0.1.
^^ Agoraphobia without panic, social phobia, specific phobia, panic disorder, Post-Traumatic Stress Disorder (PTSD), Generalized Anxiety Disorder (GAD), and separation anxiety disorder.

-	-	Total Sample (n=939) Multivariate				Males (n=404) Multivariate				Females (n=535) Multivariate			
-	-	OR	95% Confidence Limits		P-value	OR	95% Confidence Limits		P-value	OR	95% Confidence Limits		P-value
War CTEs	Active War Exposure	-	-	-	-	1.68	0.41	6.88	0.467	-	-	-	-
	General War Exposure	1.62	1.09	2.4	0.017*	1.89	0.89	4	0.096~	1.49	0.93	2.38	0.095~
	Direct Personal Trauma	-	-	-	-	2.36	0.57	9.78	0.235	-	-	-	-
	Trauma to Others	-	-	-	-	-	-	-	-	-	-	-	-

contd.....

-	-	Total Sample (n=939) Multivariate				Males (n=404) Multivariate				Females (n=535) Multivariate			
-	-	OR	95% Confidence Limits		P-value	OR	95% Confidence Limits		P-value	OR	95% Confidence Limits		P-value
Non War CTEs	Direct Personal Trauma	0.82	0.35	1.94	0.654	-	-	-	-	1.03	0.38	2.82	0.951
	Other Personal Trauma	-	-	-	-	-	-	-	-	-	-	-	-
	Trauma to Others	2.27	1.41	3.67	0.001*	2.35	0.98	5.65	0.054~	2.18	1.23	3.86	0.008*
	Caused Harm to Someone	-	-	-	-	-	-	-	-	-	-	-	-
	Neglect & Abuse	2.02	0.99	4.09	0.051~	1.04	0.27	4.11	0.949	2.19	0.93	5.18	0.073~
	Parental Loss	-	-	-	-	1.94	0.86	4.37	0.111	-	-	-	-
	Parent Psychopathology	2.23	1.34	3.74	0.002*	2.7	1.04	6.99	0.041*	1.91	1.03	3.356	0.041*
	Other Adversities within the Family	-	-	-	-	-	-	-	-	-	-	-	-
-	Sex (M *vs* F)	0.3	0.2	0.46	<0.001*	-	-	-	-	-	-	-	-

Note: *: p<0.05.
~: p<0.1.
^^ Agoraphobia without panic, social phobia, specific phobia, panic disorder, Post-Traumatic Stress Disorder (PTSD), Generalized Anxiety Disorder (GAD), and separation anxiety disorder.

© 2023 The Author(s). Published by Bentham Science Publisher.

This is an open access article distributed under the terms of the Creative Commons Attribution 4.0 International Public License (CC-BY 4.0), a copy of which is available at: https://creativecommons.org/licenses/by/4.0/legalcode. This license permits unrestricted use, distribution, and reproduction in any medium, provided the original author and source are credited.
